# Supplementary material for: Unifying non-Markovian dynamics and agent heterogeneity in scalable stochastic networks
Source: Nat Commun. 2026 Mar 2;17:3345. doi: 10.1038/s41467-026-69817-y (PMC13066373; doi:10.1038/s41467-026-69817-y)
Supplement: Supplementary file 1 — Supplementary Information [file 41467_2026_69817_MOESM1_ESM.pdf]

Supplementary Materials

Unifying Non-Markovian Dynamics and Agent Heterogeneity in Scalable Stochastic Networks

Aurelien Pelissier[1,2,3], Miroslav Phan[1,2], Didier Le Bail[4], Niko Beerenwinkel[2], Marıa Rodrıguez Martınez[1,5]

[1]IBM Research Europe, 8803 Ruschlikon, Switzerland
[2]Department of Biosystems Science and Engineering, ETH Zurich, 4058 Basel, Switzerland
[3]Institute of Computational Life Sciences, Zurich University of Applied Sciences (ZHAW), 8820, Wadenswil, Switzerland
[4]Centre de Physique Théorique (CPT), Aix-Marseille University, CNRS, 13009 Marseille, France
[5]Department of Biomedical Informatics & Data Science, Yale School of Medicine, New Haven, CT, United States
[†]Corresponding author maria.rodriguezmartinez@yale.edu.

A Probability density functions in the MOSAIC framework

A.1 Parametrization of inter-event time distributions

For consistency with other works in the literature of stochastic simulations, we parameterize processes using their mean $\frac{1}{\lambda_0}$, where λ_0 would be the instantaneous rate if the distribution was exponential. Additionally, some distributions require a second parameter to describe their *shape*. For the normal and log-normal distribution, we use γ, a scale-free standard deviation defined as the standard deviation over the mean. For the gamma and Weibull distributions we use α, the shape parameter in their standard parametrization. Thus, all IEDs in this article are parametrized with two variables, (λ_0, α) or (λ_0, γ).

Normal distribution. The normal distribution is typically parametrized by the mean μ and the standard deviation σ, as follows:

$$normal(t;\mu,\sigma) = \frac{1}{\sigma\sqrt{2\pi}} \exp\left(-\frac{1}{2}\left(\frac{t-\mu}{\sigma}\right)^2\right). \quad [24]$$

The parameters μ and σ can be chosen such as the mean inter-event time distribution $1/\lambda_0$ with:

$$\mu = \frac{1}{\lambda_0} \text{ and } \sigma = \frac{\gamma}{\lambda_0}$$

Where γ corresponds to the ratio between the standard deviation and the mean, representing a scale-free standard deviation. The alternative parametrization of the distribution for MOSAIC is then given by

$$\boxed{normal(t;\lambda_0,\gamma) = \frac{\lambda_0}{\gamma\sqrt{2\pi}} \exp\left(-\frac{1}{2}\left(\frac{\lambda_0 t-1}{\gamma}\right)^2\right).} \quad [25]$$

Log-normal distribution. The log-normal distribution is typically parametrized by μ and σ, as follows:

$$log\text{-}normal(t;\mu,\sigma) = \frac{1}{t\sigma\sqrt{2\pi}} \exp\left(-\frac{(\log(t)-\mu)^2}{2\sigma^2}\right), \quad [26]$$

The mean and variance are given by:

$$mean = \exp\left(\mu + \frac{\sigma^2}{2}\right),$$
$$variance = \left[\exp\left(\sigma^2\right) - 1\right] \exp\left(2\mu + \sigma^2\right).$$

If we want the mean inter-event time distribution to be $1/\lambda_0$, we chose μ and σ as follows:

$$\mu = \log\left(\frac{1}{\lambda_0\sqrt{1+\gamma^2}}\right) \text{ and } \sigma = \sqrt{\log\left(1+\gamma^2\right)}.$$

Where, as with the normal distribution, γ corresponds to the ratio between the standard deviation and the mean, and thus, it is scale invariant. The alternative parametrization of the distribution for MOSAIC is then given by

$$\boxed{\textit{log-normal}(t;\lambda_0,\gamma) = \frac{1}{t\sqrt{2\pi\log(1+\gamma^2)}}\exp\left(-\frac{\left(\log(t)+\log\left(\lambda_0\sqrt{1+\gamma^2}\right)\right)^2}{2\log(1+\gamma^2)}\right),} \qquad [27]$$

Gamma distribution. The gamma distribution admits 2 constants, α and β:

$$gamma(t;\alpha,\beta) = \frac{\beta^\alpha}{\Gamma(\alpha)}t^{\alpha-1}e^{-\beta t}. \qquad [28]$$

Mean and variance can be computed to be:

$$\begin{aligned} mean =& \frac{\alpha}{\beta}, \\ variance =& \frac{\alpha}{\beta^2}. \end{aligned}$$

The parameter β can be chosen such as the mean inter-event time distribution is $1/\lambda_0$:

$$\beta = \alpha\lambda_0 .$$

Thus, we parametrize the gamma distribution as

$$\boxed{gamma(t;\lambda_0,\alpha) = \frac{(\alpha\lambda_0)^\alpha}{\Gamma(\alpha)}t^{\alpha-1}e^{-\alpha\lambda_0 t}.} \qquad [29]$$

We note that in this case the ratio between the standard deviation and the mean is related the the shape parameter α with:

$$\gamma = \frac{1}{\sqrt{\alpha}},$$

Weibull distribution. The Weibull distribution [1] can be parametrized with constants λ and α as follows:

$$Weibull(t;\lambda,\alpha) = \frac{\alpha}{\lambda}\left(\frac{t}{\lambda}\right)^{\alpha-1}e^{-(t/\lambda)^\alpha}. \qquad [30]$$

The mean and variance are:

$$\begin{aligned} mean =& \lambda\,\Gamma\left(1+\frac{1}{\alpha}\right), \\ variance =& \lambda^2\left[\Gamma\left(1+\frac{2}{\alpha}\right)-\Gamma^2\left(1+\frac{1}{\alpha}\right)\right]. \end{aligned}$$

If t represents a "time-to-failure", the Weibull distribution gives a distribution for which the failure rate is proportional to a power of time. An alternative parametrisation often found in text books is $\lambda = \left(\frac{\alpha}{\beta}\right)^{\frac{1}{\alpha}}$, under which the PDF of the Weibull distribution becomes:

$$Weibull\,(t;\beta,\alpha) = \beta t^{\alpha-1}\times\exp\left(-\frac{\beta t^\alpha}{\alpha}\right). \qquad [31]$$

To make the mean inter event time distribution equal to $1/\lambda_0$, β has to be chosen as follows:

$$\beta = \alpha\left[\lambda_0\Gamma\left(\frac{\alpha+1}{\alpha}\right)\right]^\alpha.$$

Thus, we parametrize the gamma distribution as

$$\boxed{Weibull\,(t;\lambda_0,\alpha) = \alpha\left[\lambda_0\Gamma\left(\frac{\alpha+1}{\alpha}\right)\right]^{\alpha} t^{\alpha-1}\times\exp\left(-\frac{\alpha\left[\lambda_0\Gamma\left(\frac{\alpha+1}{\alpha}\right)\right]^{\alpha}t^{\alpha}}{\alpha}\right).} \quad [32]$$

We note that with this choice, the ratio between the standard deviation and the mean is related to α with:

$$\gamma = \frac{\Gamma\left(\frac{\alpha+2}{\alpha}\right)}{\Gamma^2\left(\frac{\alpha+1}{\alpha}\right)} - 1\,.$$

Cauchy distribution. The Cauchy distribution admits two parameters, μ and σ:

$$Cauchy(t;\mu,\sigma) = \frac{1}{\pi\sigma\left[1+\left(\frac{t-\mu}{\sigma}\right)^2\right]}\,. \quad [33]$$

The Cauchy distribution represents the distribution of the ratio of two independent and normally distributed random variables with mean zero. The mean and variance are undefined, as the integrals necessary to compute these values do not exist[1]. Intuitively, this happens because extremely large number can be drawn with non-zero probability. Nevertheless, we can choose the parameter μ as the inverse of the median inter event time $1/\lambda_0$:

$$\mu = \frac{1}{\lambda_0}\,.$$

Similarly, we can define γ as the *analogue* of ratio between the standard deviation and the mean, which is scale invariant:

$$\sigma = \frac{\gamma}{\lambda_0}\,.$$

Thus, we parametrize the Cauchy distribution as

$$\boxed{Cauchy(t;\lambda_0,\gamma) = \frac{1}{\pi\frac{\gamma}{\lambda_0}\left[1+\left(\frac{\lambda_0 t-1}{\gamma}\right)^2\right]}\,.} \quad [34]$$

Pareto distribution. The Pareto distribution admits two parameters, μ and σ:

$$Pareto(t,\mu,\alpha) = \begin{cases}\frac{\alpha\mu^{\alpha}}{t^{\alpha+1}} & t\geq\mu\\ 0 & t<\mu\end{cases} \quad [35]$$

As with the Cauchy distribution, the Pareto distribution does not always have a finite mean, so we choose the parameter μ such as its median equals $1/\lambda_0$:

$$\mu = \frac{1}{\lambda_0}2^{-1/\alpha}\,. \quad [36]$$

Thus, we parametrize the Pareto distribution as

$$\boxed{Pareto(t,\lambda_0,\alpha) = \begin{cases}\frac{\alpha}{t^{\alpha+1}}\cdot\frac{1}{2\lambda_0^{\alpha}} & t\geq\frac{1}{\lambda_0}2^{-1/\alpha}\\ 0 & t<\frac{1}{\lambda_0}2^{-1/\alpha}\end{cases}} \quad [37]$$

[1]The integral associated with the mean, $\int_{-\infty}^{\infty} xf(x)\,dx$, does not exist. This can be proven, for instance, by noticing that $\lim_{a\to\infty}\int_{-a}^{a} xf(x)\,dx$ and $\lim_{a\to\infty}\int_{-2a}^{a} xf(x)\,dx$ converge to different values. Similar arguments can be used to show that the variance does not exist either.

A.2 Relationship between probability density functions and instantaneous rates

We consider the survival distribution function (SDF) $\Psi(t)$ of a renewal process:

$$\Psi(t) = \int_t^{\infty} \psi(\tau)\,\mathrm{d}\tau \qquad [38]$$

and its relationship with the probability distribution function (PDF) $\psi(t) = -\frac{d\Psi(t)}{dt}$. Using the definition of the instantaneous rate function:

$$\lambda(t) = \frac{\psi(t)}{\Psi(t)}, \qquad [39]$$

we can describe the time evolution of $\Psi(t)$ as a first order homogeneous differential equation:

$$\lambda(t)\,\Psi(t) + \Psi'(t) = 0. \qquad [40]$$

The general solution is easily written as [2]:

$$\Psi(t) = K \exp\left(-\int_0^t \lambda(\tau)d\tau\right), \qquad [41]$$

where $K \in \mathbb{R}$ is an integration constant. However, since by definition $\Psi(0) = 1$, we conclude that $K = 1$. The PDF of that process can now by computed as follows:

$$\psi(t) = -\frac{d\Psi}{dt}(t) = \lambda(t)\,\exp\left(-\int_0^t \lambda(\tau)d\tau\right) \qquad [42]$$

Note that $\Psi(t)$ verifies the additional normalization condition $\Psi(\infty) = 0$. This implies:

$$\begin{aligned}\Psi(\infty) &= \exp\left(-\int_0^{\infty} \lambda(\tau)d\tau\right) = 0 \\ \Rightarrow & \int_0^{\infty} \lambda(\tau)d\tau = \infty \\ .\end{aligned} \qquad [43]$$

This means that $\lambda(t)$ has to be chosen such as its definite integral from 0 to ∞ is infinite, otherwise $\psi(t)$ and $\Psi(t)$ do not represent a renewal process.

Examples

In general, the instantaneous rate for any distribution can be computed as $\lambda(t) = \frac{PDF(t)}{SDF(t)}$, where the survival distribution function (SDF) is related to the cumulative distribution function (CDF) according to $SDF = 1 - CDF$.
We provide here a few examples of instantaneous rate functions and their associated PDFs:

- $\lambda(t) = a_0$ leads to $PDF = a_0 \times \exp(-a_0 t)$ and $SDF = \exp(-a_0 t)$, which represent an exponential distribution.
- $\lambda(t) = \beta t^{\alpha-1}$ leads to $PDF = \beta t^{\alpha-1} \times \exp\left(-\frac{\beta t^{\alpha}}{\alpha}\right)$ and $SDF = \exp\left(-\frac{\beta t^{\alpha}}{\alpha}\right)$, associated with the Weibull distribution [1].
- $\lambda(t) = \dfrac{c^2 t}{1+ct}$ leads to $PDF = c^2 t \times \exp\big(-ct\big)$ and $SDF = (1+ct) \times \exp\big(-ct\big)$.
- Many important distributions do not have a simple analytic form for the instantaneous rate. For instance, the normal distribution, $PDF = \frac{1}{\sigma\sqrt{2\pi}} e^{-\frac{1}{2}\left(\frac{t-\mu}{\sigma}\right)^2}$ and $SDF = \frac{1}{2}\left[1 - \mathrm{erf}\left(\frac{t-\mu}{\sigma\sqrt{2}}\right)\right]$, with *erf* being the error function [3], results in an instantaneous rate that cannot be expressed in terms of basic functions. An approximation is however possible at large times, where the instantaneous rate asymptotically approximates a linear function $\lambda(t) \approx \frac{t-\mu}{\sigma^2}$.

In Figure S1B, we show that the normal, Weibull ($\alpha \geq 1$) and gamma ($\alpha \geq 1$) distributions have monotonically increasing rates, while the Cauchy and log-normal distributions exhibit a maximum.

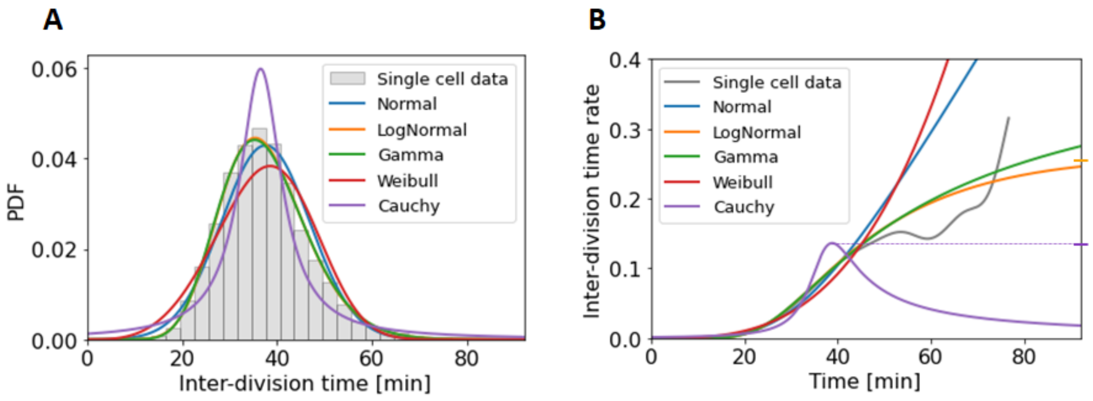

Figure S1: (A) PDF of several distributions typically used to represent biochemical waiting times, where the parameters have been chosen to fit the measured inter-division time of *Bacillus subtilis* [4] at constant temperature. (B) Although the PDFs are relatively similar, the instantaneous rates show markedly different behaviors as a function of time. The single-cell instantaneous rate was estimated with a Gaussian kernel density estimator. When finite, the maximum instantaneous rates are displayed on the right of the figure.

B Modeling of Stochastic Agents with Individual Complexity

We consider N Poisson processes running in parallel, each with their respective reaction rate λ_j $(1 \leq j \leq N)$, and denote by $a_0 = \sum \lambda_j$ the sum of the individual rates. The standard Gillespie (SG) algorithm is a popular stochastic simulation framework that can generate statistically correct trajectories of a stochastic equation system. The algorithm assumes that the reaction rates are known and constant, under which assumption, a trajectory can be obtained using the following iterative rules (see [5] for the full derivation):

(i) Draw $u \in \mathcal{U}^{[0,1]}$ a random variate from the uniform density on the interval $[0, 1]$, and compute the time increment to the next event with

$$\Delta t = \frac{\ln(1/u)}{a_0}, \quad [44]$$

(ii) Draw the process j that has produced the event with probability

$$P_j = \frac{\lambda_j}{a_0}, \quad [45]$$

In the main text, we introduced MOSAIC with a Rejection Gillespie approach, that is statistically exact in the limit of $\Delta t \to 0$. We provide the algorithm here again for reference.

MOSAIC Algorithmic procedure. Let t_j denote the time elapsed since the last event of the jth process $(1 \leq j \leq N)$, and $\lambda_j(t_j)$ its time-dependent rate. Each iteration of MOSAIC proceeds in four steps:

(i) *Set the global maximum rate.* Choose $\lambda_{\max}$ such that

$$\lambda_{\max} \geq \max_{1 \leq j \leq N} \lambda_j(t_j). \quad [46]$$

(ii) *Advance the time.* Draw $u \sim \mathcal{U}[0, 1]$ and set

$$\Delta t = \frac{\ln(1/u)}{N \cdot \lambda_{\max}}. \quad [47]$$

(iii) *Select a candidate process.* Choose j uniformly at random:

$$p_j = \frac{1}{N}. \quad [48]$$

(iv) *Accept or reject the event.* Accept with probability

$$p_{\text{accept}} = \frac{\lambda_j(t_j)}{\lambda_{\max}}, \quad [49]$$

in which case the reactants are updated. If rejected, the event is empty (no population change), but time still advances by Δt.

Below, we provide the proof, which we decompose into two independent subparts. First, we show that the introduction of rejected steps in the Gillespie model is mathematically equivalent to the standard Gillespie (SG) algorithm. Second, we show that locally considering each process as Poissonian during the time step Δt at each Gillespie iteration yields statistically exact results for non-Markovian simulations when $\Delta t \to 0$. We note that both of these aspects were already proven separately in reference [6] and [7], respectively. Here we put them together for the reader's convenience. As the rejection framework allows for the arbitrary reduction of Δt by increasing $\lambda_{\max}$, MOSAIC is exact in the limit $\lambda_{\max} \to \infty$ or $N \to \infty$.

B.1 Proof for the Rejection-based Gillespie Algorithm

The rejection-based Gillespie algorithm follows the same distribution as the Standard Gillespie (SG) if and only if (iff):

(i) The reaction R_j occurs with probability $P = \lambda_j / a_0$.
(ii) The Δt time increment follows the same exponential distribution as in SG, i.e. $f_{\Delta t}(x) = a_0 \cdot \exp(-a_0 x)$.

(i) Reaction R_j accepted with the same probability as in SG

We define $p_{\text{accept}}(R_j)$ as the joint probability of R_j being first selected and then accepted, $\lambda_{\max}$ as the upper propensity bound for all reactions, and $a_{0,\max} = N\lambda_{\max}$. We can write:

$$p_{\text{accept}}(R_j) = \frac{\lambda_{\max}}{a_{0,\max}} \times \frac{\lambda_j}{\lambda_{\max}} = \frac{\lambda_j}{a_{0,\max}}$$

We then denote by $p_{\text{accept}}(R)$ the probability of any reaction being accepted:

$$p_{\text{accept}}(R) = \frac{a_0}{a_{0,\max}}.$$

Finally, we write the conditional probability of reaction R_j being accepted given that some reaction had been accepted as:

$$p_{\text{accept}}(R_j \mid R) = \frac{p_{\text{accept}}(R_j)}{p_{\text{accept}}(R)} = \left(\frac{\lambda_j}{a_{0,\max}}\right) / \left(\frac{a_0}{a_{0,\max}}\right) = \frac{\lambda_j}{a_0},$$

in agreement with SG framework.

(ii) The time increment Δt follows an exponential PDF

We denote by k the number of trials until the reaction is accepted (thus, there are $k-1$ rejections until success), with time being advanced by an increment of $\Delta t = -\ln(u)/a_{0,\max}$ after each attempt. It follows that after k attempts, the time increment is:

$$\Delta t = -\frac{1}{a_{0,\max}} \ln\left(\prod_{i=1}^{k} u_i\right) \qquad [50]$$

which corresponds to an Erlang distribution with parameters k and $a_{0,\max}$. This distribution represents the time elapsed until the kth event of a Poisson process with rate $a_{0,\max}$. In addition, k is geometrically distributed with probability $p_{\text{accept}}(R)$, i.e.

$$P(X = k) = (1 - p_{\text{accept}}(R))^{k-1} \times p_{\text{accept}}(R).$$

As the PDF for Δt can be expressed as the derivative of its CDF, we can write

$$\begin{aligned} f_{\Delta t}(x) &= \frac{d}{dx} F_{\Delta t}(x) \\ &= \frac{d}{dx} P(\Delta t \leq x), \end{aligned}$$

where $P(\Delta t \leq x)$ can be partitioned for values of k:

$$\begin{aligned} &= \frac{d}{dx} \sum_{k=1}^{\infty} P(\Delta t \leq x \mid X = k) P(X = k) \\ &= \frac{d}{dx} \sum_{k=1}^{\infty} P(\Delta t \leq x \mid X = k) \left(1 - \frac{a_0}{a_{0,\max}}\right)^{k-1} \frac{a_0}{a_{0,\max}} \end{aligned}$$

and as shown in Eq. 50, the distribution of Δt parametrized by k follows an Erlang distribution:

$$
\begin{aligned}
&= \sum_{k=1}^{\infty} \frac{d}{dx} F_{\mathrm{Erlang}(k,\lambda_{0,\max})} \left(1 - \frac{a_0}{a_{0,\max}}\right)^{k-1} \frac{a_0}{a_{0,\max}} \\
&= \sum_{k=1}^{\infty} f_{\mathrm{Erlang}(k,\lambda_{0,\max})} \left(1 - \frac{a_0}{a_{0,\max}}\right)^{k-1} \frac{a_0}{a_{0,\max}} \\
&= \sum_{k=1}^{\infty} \frac{a_{0,\max}^k \cdot x^{k-1} \cdot \exp(-a_{0,\max} x)}{(k-1)!} \cdot \left(\frac{a_{0,\max} - a_0}{a_{0,\max}}\right)^{k-1} \cdot \frac{a_0}{a_{0,\max}} \\
&= a_0 \exp(-a_{0,\max} x) \sum_{k=1}^{\infty} \frac{(a_{0,\max} - a_0)^{k-1} \cdot x^{k-1}}{(k-1)!} \\
&= a_0 \exp(-a_{0,\max} x) \cdot \exp(x \cdot (a_{0,\max} - a_0)) \\
&= a_0 \cdot \exp(-a_0 x).
\end{aligned}
$$

Hence, in Rejection Gillespie, Δt follows the same exponential distribution as in the SG.

B.2 Proof for the Non-Markovian Gillespie Algorithm (nMGA)

In this section, we summarize the proof given by Boguna [7], and consider a second-order approximation of their algorithm. We consider N renewal processes running in parallel, and denote by t_j the time elapsed since the last event of the jth process ($1 \leq j \leq N$). We denote by $\psi_j(t_j)$ the probability density function of inter-event times for the jth process, and by

$$\Psi_j(t_j) = \int_{t_j}^{\infty} \psi_j(\tau)\, \mathrm{d}\tau \,, \qquad [51]$$

the survival function of the jth process, i.e., the probability that the inter-event time is larger than t_j. We also set

$$\Phi(\Delta t \mid \{t_j\}) = \prod_{j=1}^{N} \frac{\Psi_j(t_j + \Delta t)}{\Psi_j(t_j)} \qquad [52]$$

which is the probability that no process generates an event for time Δt [5]. Then in the non-Markovian Gillespie algorithm (nMGA), the time until the next event, Δt, is computed by solving $\Phi(\Delta t \mid \{t_j\}) = u$, where $u \in \mathcal{U}^{[0,1]}$ is a random variate drawn from the uniform density on the interval $[0, 1]$. This can be time-consuming for some distributions [7]. In the limit of a large number of processes $N \to \infty$, we can simplify the numerical computation of the time Δt needed in the algorithm. We start by rewriting the function $\Phi(\Delta t | tj)$ as:

$$\Phi(\Delta t \mid \{t_j\}) = \exp\left[-\sum_{j=1}^{N} \ln\left(\frac{\Psi_j(t_j)}{\Psi_j(t_j + \Delta t)}\right)\right] \qquad [53]$$

The sum within the exponential function is a sum of N monotonously increasing functions of Δt. Therefore, when $N \to \infty$, the survival probability $\Phi(\Delta t \mid \{t_j\})$ is close to zero everywhere except when $\Delta t \sim 0$. Hence, we only need to consider $\Phi(\Delta t \mid \{t_j\})$ around $\Delta t = 0$. In this neighborhood, we can perform a Taylor expansion for small Δt, namely, $\Psi_j(t_j + \Delta t) \approx \Psi_j(t_j) - \psi_j(t_j)\Delta t + O(\Delta t^2)$. Plugging this expression into Eq. 53, using the approximation $\ln(1 + x) \approx x + O(x^2)$ and $1/(1 - x) \approx 1 + x + O(x^2)$ when $x \to 0$, we can write:

$$
\begin{aligned}
\Phi(\Delta t \mid \{t_j\}) &= \exp\left[-\sum_{j=1}^{N} \ln \frac{\Psi_j(t_j)}{\Psi_j(t_j + \Delta t)}\right] \\
&\approx \exp\left[-\sum_{j=1}^{N} \ln \frac{\Psi_j(t_j)}{\Psi_j(t_j) - \psi_j(t_j)\Delta t + O(\Delta t^2)}\right] \qquad [54] \\
&\approx \exp\left[-\Delta t \left(\sum_{j=1}^{N} \lambda_j(t_j)\right) + O\left(\Delta t^2\right)\right],
\end{aligned}
$$

where the instantaneous rate λ_j is defined as:

$$\lambda_j(t_j) = \frac{\psi_j(t_j)}{\Psi_j(t_j)}.$$

With this approximation, the time until the next event is determined by

$$\Phi\left(\Delta t \mid \{t_j\}\right) \approx \exp\left[-\Delta t\left(\sum_{j=1}^{N} \lambda_j\left(t_j\right)\right)\right]. \quad [55]$$

Denoting by u a uniform random variable sampled from $[0,1]$ to represent $\Phi\left(\Delta t \mid \{t_j\}\right)$, we can approximate Δt as follows:

$$\Delta t \approx \frac{\ln(1/u)}{\sum_{j=1}^{N} \lambda_j(t_j)}. \quad [56]$$

We note that when $\lambda_j(t_j)$ depends on time, the uniform random variable u may not directly correspond to the distribution $\Phi\left(\Delta t \mid \{t_j\}\right)$. However, the approximation remains valid within a small neighborhood around $\Delta t = 0$ and when $\lambda_j(t_j)$ changes slowly with time. By eliminating the time dependency, i.e. setting $\lambda_j\left(t_j\right) = \lambda_j$, we recover the SG algorithm, where Δt is exponentially distributed. In the limit where $\lambda_j(t_j)$ changes slowly with time, Δt is still approximately exponentially distributed. nMGA exploits this approximation and locally treats each process as Poissonian during the time step Δt.

Second-order approximation

We can also consider a quadratic expansion of Eq. 53, which results in a second-order approximation of the time interval Δt. To do so, we first expand up to second order $\Psi_j\left(t_j + \Delta t\right)$:

$$\Psi_j\left(t_j + \Delta t\right) \approx \Psi_j\left(t_j\right) - \psi_j\left(t_j\right)\Delta t - \psi_j'\left(t_j\right)\Delta t^2/2 + O\left(\Delta t^3\right), \quad [57]$$

where we remind the reader that $\Psi_j' = -\psi_j$. Additionally, the instantaneous rate $\lambda_j = \psi_j/\Psi_j$ is related to its derivative λ_j' through the relation:

$$\lambda_j' = \left(\frac{\psi_j}{\Psi_j}\right)' = \frac{\psi_j'\Psi_j - \psi_j\Psi_j'}{\Psi_j^2} = \frac{\psi_j'}{\Psi_j} + \left(\frac{\psi_j}{\Psi_j}\right)^2 = \frac{\psi_j'}{\Psi_j} + \lambda_j^2. \quad [58]$$

Plugging this expression into Eq. 53, using the approximation $1/(1-x) = 1 + x + x^2 + O\left(x^3\right)$ and $\ln(1+x) = x - x^2/2 + O\left(x^3\right)$ for $x \to 0$, we obtain:

$$\begin{aligned}
\Phi\left(\Delta t \mid \{t_j\}\right) &\approx \exp\left[-\sum_{j=1}^{N} \ln \frac{1}{1 - \lambda_j\left(t_j\right)\Delta t - \left(\lambda_j'(t_j) - \lambda_j^2(t_j)\right)\frac{\Delta t^2}{2} + O\left(\Delta t^3\right)}\right] \\
&\approx \exp\left[-\sum_{j=1}^{N} \ln\left(1 + \lambda_j\left(t_j\right)\Delta t + \left(\lambda_j'(t_j) + \lambda_j^2(t_j)\right)\frac{\Delta t^2}{2} + O\left(\Delta t^3\right)\right)\right] \\
&\approx \exp\left[-\sum_{j=1}^{N}\left(\lambda_j\left(t_j\right)\Delta t + \lambda_j'(t_j)\frac{\Delta t^2}{2} + O\left(\Delta t^3\right)\right)\right]
\end{aligned} \quad [59]$$

Solving $\Phi\left(\Delta t \mid \{t_j\}\right) = u$ to determine the next time increment Δt, we get a quadratic equation for which we take the positive solution:

$$\Delta t \approx \frac{-\sum_{j=1}^{N} \lambda_j(t_j) + \sqrt{\left(\sum_{j=1}^{N} \lambda_j(t_j)\right)^2 + 2\left(\sum_{j=1}^{N} \lambda_j'(t_j)\right)\cdot \ln(1/u)}}{\left(\sum_{j=1}^{N} \lambda_j'(t_j)\right)}. \quad [60]$$

Once again, Eq. 60 makes explicit that Δt is no longer exponentially distributed when $\lambda_j'(t_j) \neq 0$, and hence, the rejection framework described in the first part cannot be applied with the second-order approximation of nMGA.

B.3 Quantifying the errors of nMGA and MOSAIC

Let us derive the error for both nMGA and MOSAIC. As a reminder, at each time step, both nMGA and MOSAIC utilize the first-order approximation of the survival distribution function to generate the next event with

$$\Psi_j\left(t_j+\Delta t\right)=\Psi_j\left(t_j\right)-\psi_j\left(t_j\right)\Delta t+O\left(\psi_j'(t_j)\Delta t^2\right), \tag{61}$$

from which the probability that no process generates an event for time Δt with (Eq. 52) is computed from

$$\Phi\left(\Delta t \mid \{t_j\}\right)=\prod_{j=1}^{N}\frac{\Psi_j\left(t_j+\Delta t\right)}{\Psi_j\left(t_j\right)}, \tag{62}$$

where N is the total number of processes being considered. The error in this approximation is given by the difference between the actual value $\Phi\left(\Delta t \mid \{t_j\}\right)$ and the approximated value derived from the first term of the Taylor expansion. According to Taylor's theorem, and looking at the first and second order expression of Eq.59, we write the error term for the first-order approximation of $\ln\left[\Phi\left(\Delta t \mid \{t_j\}\right)\right]$ as the second-order remainder [8]:

$$R_2^{\ln\Phi}=\sum_{j=1}^{N}\lambda_j'(t_j)\frac{\Delta t^2}{2}+O(\Delta t^3), \tag{63}$$

where $\lambda_j'(t_j)$ is the derivative of the instantaneous rate function at time t_j.

Error of nMNGA. Plugging in the value of Δt of nMGA (Eq.56), we estimate the error $R_2^{\ln\Phi}$ in nMGA per time step as

$$\left(R_2^{\ln\Phi}\right)_{\text{nMGA}}\sim\sum_{j=1}^{N}\frac{\lambda_j'(t_j)}{2}\left(\frac{\ln(1/u)}{\sum_{i=1}^{N}\lambda_i(t_i)}\right)^2\sim\frac{\ln^2(1/u)}{2N}\cdot\frac{\langle\lambda'\rangle}{\langle\lambda\rangle^2}, \tag{64}$$

where we have introduced $\langle\lambda\rangle$ and $\langle\lambda'\rangle$, the average rate and the average derivative of the rates respectively, i.e.:

$$\langle\lambda\rangle=\frac{1}{N}\sum_{j=1}^{N}\lambda_j(t_j)\ \text{ and }\ \langle\lambda'\rangle=\frac{1}{N}\sum_{j=1}^{N}\lambda_j'(t_j)\,. \tag{65}$$

Error of MOSAIC. Using instead MOSAIC's time increment (Eq.2),

$$\Delta t=\frac{\ln(1/u)}{N\lambda_{\max}}, \tag{66}$$

we obtain MOSAIC's error estimate:

$$\left(R_2^{\ln\Phi}\right)_{\text{MOSAIC}}\sim\frac{\ln^2(1/u)}{2N}\cdot\frac{\langle\lambda'\rangle}{\lambda_{\max}^2}\,. \tag{67}$$

We note that Eqs. 64 and 67 depend on $\ln^2(1/u)$, which does not have an upper bound so the error in a particular iteration can become arbitrarily large. However, we can write the expected error by substituting $\ln^2(1/u)$ with $\mathbb{E}[\ln^2(1/u)]=\int_0^\infty t^2e^{-t}\,dt=2$, where

$$\mathbb{E}\left[\left(R_2^{\ln\Phi}\right)_{\text{nMGA}}\right]\sim\frac{\langle\lambda'\rangle}{N\cdot\langle\lambda\rangle^2}\quad\text{and}\quad\mathbb{E}\left[\left(R_2^{\ln\Phi}\right)_{\text{MOSAIC}}\right]\sim\frac{\langle\lambda'\rangle}{N\cdot\lambda_{\max}^2}\,. \tag{68}$$

Here, we make several noteworthy observations. First, in both nMGA and MOSAIC, the error is directly proportional to the first derivative of the rate function, so both methods yield exact results for the exponential distributions where $\lambda_j'(t_j)=0$. In contrast, distributions with rapidly changing rates, such as the Weibull distribution with a high shape parameter, will exhibit significantly higher errors compared to distributions with more gradually varying rates, such as long-tailed distributions like Cauchy or Pareto.

Then, the primary difference between the two methods lies in the denominators of the expected error, which dictate how errors scale. Specifically, the error in nMGA scales with $1/\langle\lambda\rangle^2$, while MOSAIC's scales with $1/\lambda_{\max}^2$. Consequently, nMGA is particularly prone to large errors when agents are initialized with low instantaneous rates, as $\langle\lambda\rangle$ can be very small at the start of the simulation. This issue is not encountered in MOSAIC, as $\lambda_{\max}$ can be chosen independently, providing greater flexibility and accuracy.

Error in the time increment Δt. The errors derived above reflect the uncertainty in estimating $\Phi(\Delta t)$ at a specific time step, the probability that no events occur within the interval Δt. To quantify the error in the time step increment itself, we consider the sensitivity of Δt to variations in $\ln \Phi(\Delta t)$. This error propagation can be determined by applying the chain rule [9]:

$$\mathrm{Error}[\Delta t] \sim \left| \frac{\partial \Delta t}{\partial \ln \Phi(\Delta t)} \right| \cdot \mathrm{Error}[\ln \Phi(\Delta t)], \tag{69}$$

where the term $\frac{\partial \Delta t}{\partial \ln \Phi(\Delta t)}$ represents how sensitive the time increment is to changes in $\ln \Phi(\Delta t)$. Here, we have ignored the sign, focusing only on the magnitude of the error. To analytically derive the error of Δt in MOSAIC, we differentiate

$$\Phi_{\text{MOSAIC}}(\Delta t) \sim \exp\left[-\Delta t N \lambda_{\max}\right] \tag{70}$$

and find that

$$\left| \frac{\partial \ln \Phi_{\text{MOSAIC}}(\Delta t)}{\partial \Delta t} \right| \sim N\lambda_{\max}. \tag{71}$$

The error in Δt can thus be propagated as

$$\mathrm{Error}[\Delta t_{\text{MOSAIC}}] \sim \frac{1}{N\lambda_{\max}} \cdot \mathrm{Error}[\ln \Phi_{\text{MOSAIC}}(\Delta t)], \tag{72}$$

which we also write as

$$\mathrm{Error}[\Delta t_{\text{MOSAIC}}] \sim \frac{1}{N\lambda_{\max}} \cdot \left(R_2^{\ln \Phi} \right)_{\text{MOSAIC}}. \tag{73}$$

Applying the same approach for nMGA, we write

$$\Phi_{\text{nMGA}}(\Delta t) \sim \exp\left[-\Delta t \left(\sum_{j=1}^{N} \lambda_j(t_j) \right) \right] \sim \exp\left[-\Delta t N \langle \lambda \rangle\right] \tag{74}$$

and obtain

$$\mathrm{Error}[\Delta t_{\text{nMGA}}] \sim \frac{1}{N\langle \lambda \rangle} \cdot \left(R_2^{\ln \Phi} \right)_{\text{nMGA}}. \tag{75}$$

Accumulated error in the IED. In a stochastic simulation, errors accumulate across multiple time steps, denoted here as n. The total error in the simulated IED can be expressed as the sum of errors over all time steps:

$$\mathrm{Error}[\mathrm{IED}] = \sum_{i=0}^{n} \mathrm{Error}[\Delta t_i] \sim n \cdot \mathbb{E}\Big[\mathrm{Error}[\Delta t]\Big]. \tag{76}$$

In the case of the standard Gillespie algorithm and nMGA, the number of time steps required to simulate a system for a fixed time T_{end} scales with the number of processes, N, and the mean reaction rate, $\langle \lambda \rangle$, such that $n \sim N\langle \lambda \rangle$. Consequently, the accumulated error for nMGA can be approximated as:

$$\mathrm{Error}[\mathrm{IED}_{\text{nMGA}}] \sim N\langle \lambda \rangle \cdot \mathrm{Error}[\Delta t_{\text{nMGA}}] \sim \mathbb{E}\Big[\left(R_2^{\ln \Phi} \right)_{\text{nMGA}} \Big]. \tag{77}$$

In the case of MOSAIC, where some reactions are rejected, the number of time steps increases compared to nMGA, scaling as $\sim N\lambda_{\max}$. Substituting this relation, the accumulated error for MOSAIC can be written as:

$$\mathrm{Error}[\mathrm{IED}_{\text{MOSAIC}}] \sim N\lambda_{\max} \cdot \mathrm{Error}[\Delta t_{\text{MOSAIC}}] \sim \mathbb{E}\Big[\left(R_2^{\ln \Phi} \right)_{\text{MOSAIC}} \Big]. \tag{78}$$

Here we observe that, while MOSAIC involves a greater number of time steps due to the rejection of some reactions, the total accumulated error over the course of the simulation remains comparable with nMGA, since they both equal $R_2^{\ln \Phi}$. This equivalence can be intuitively understood as a balance: the increase in the number of time steps in MOSAIC is effectively counteracted by the proportional reduction in error magnitude per time step. Substituting the expression for R_2^{Φ} derived in Eq. 68, the final expressions for the errors are:

$$\mathrm{Error}[\mathrm{IED}_{\text{nMGA}}] \sim \frac{\langle \lambda' \rangle}{N \cdot \langle \lambda \rangle^2} \quad \text{and} \quad \mathrm{Error}[\mathrm{IED}_{\text{MOSAIC}}] \sim \frac{\langle \lambda' \rangle}{N \cdot \lambda_{\max}^2}. \tag{79}$$

We point out that, for illustrative purposes, we assumed here that $\langle \lambda' \rangle$, $\langle \lambda \rangle$, and $\lambda_{\max}$ remain approximately constant throughout the simulation, a reasonable assumption for systems that quickly reach a steady state, where these parameters do not fluctuate significantly. However, this framework can also be generalized to systems with time-varying parameters. In such cases, the IED error at any given time step can be interpreted as the cumulative error that would result if the conditions (i.e., the values of $\langle \lambda' \rangle$, $\langle \lambda \rangle$, and $\lambda_{\max}$) at that specific time step were held constant and applied across the entire simulation.

Accumulated error in the population dynamics. While we successfully derived the expected error for the simulated IED, most non-Markovian studies report observables in terms of population dynamics rather than the IED, as population dynamics are generally more accessible experimentally [10, 11, 12] due to the difficulty of directly measuring IED in experimental systems. Here, we describe how errors in the time step Δt propagates into population count dynamics over the course of the simulation. We consider N processes and define the total rate as $\lambda_{\text{total}} = N\lambda_{\text{max}}$. For a given iteration, we estimate that the error in the time step propagates to the population as

$$\text{Error}[P] \sim \text{Error}[\Delta t] \cdot \frac{\lambda_{\text{total}}}{N} \sim \text{Error}[\Delta t] \cdot \lambda_{\text{max}}. \tag{80}$$

Here, we normalize the population count error by the number of processes because we are interested in the relative population rather than the absolute errors in the counts. Due to the accumulated error during the simulation, we write the deviation of the population at time t from the ground truth as

$$\Delta P(t) = \sqrt{t} \cdot \text{Error}[P], \tag{81}$$

where we have assumed that the error propagates through time like a diffusing behavior. Then, we can write the total error of the simulation as a sum through the number of total events T

$$\begin{aligned} \text{Error}[\text{POP}_{\text{MOSAIC}}] &= \sum_{k=0}^{T} \Delta P(t_k) & [82] \\ &= \text{Error}[P] \left(\sum_{k=0}^{T} \sqrt{t_k} \right) & [83] \\ &\sim \lambda_{\text{max}} T^{3/2} \cdot \text{Error}[\Delta t_{\text{MOSAIC}}] \cdot & [84] \end{aligned}$$

where the series scales approximately as $T^{3/2}$ due to the summation of $\sqrt{t}$ term. Substituting $T \sim \lambda_{\text{max}} N$ and the time step error derived under the MOSAIC framework

$$\text{Error}[\Delta t_{\text{MOSAIC}}] \sim \frac{\langle \lambda' \rangle}{N^2 \cdot \lambda_{\text{max}}^3}, \tag{85}$$

we obtain:

$$\text{Error}[\text{POP}_{\text{MOSAIC}}] \sim \sqrt{\frac{1}{N\lambda_{\text{max}}}} \langle \lambda' \rangle. \tag{86}$$

B.4 Rejection sampling allows for arbitrary approximation accuracy

In this section, we discuss the differences between nMGA [7] and MOSAIC in terms of simulation accuracy. We recall that both MOSAIC and nMGA are exact only when $\Delta t \to 0$, as they use a first-order Taylor approximation of the survival distribution function. Indeed, the survival distribution function can be Taylor approximated as follows:

$$\Psi_j\left(t_j + \Delta t\right) = \Psi_j\left(t_j\right) - \psi_j\left(t_j\right)\Delta t + O\left(\psi_j'(t_j)\Delta t^2\right). \qquad [87]$$

The approximation breaks down when the inequality $\psi_j(t_j) \gg \psi_j'(t_j)\Delta t$ is no longer verified. The main difference between MOSAIC and nMGA lies in the introduction of the rejection step. In nMGA, the time increment until the next event is computed as:

$$\Delta t\ (\text{nMGA}) = \frac{\ln(1/u)}{\sum_{j=1}^{N}\lambda_j(t_j)}, \qquad [88]$$

while in MOSAIC, the expression becomes:

$$\Delta t\ (\text{MOSAIC}) = \frac{\ln(1/u)}{N \cdot \lambda_{\max}}. \qquad [89]$$

From Eq.88, it is clear that low rates are associated with large time increments Δt, and that in such a regime, the linear approximation nMGA used to compute Δt might fail (Eq.87). Indeed, nMGA is only exact in the limit of an infinite number of processes ($N \to \infty$) where it can be assumed that $\sum_{j=1}^{N}\lambda_j(t_j) \to \infty$. For processes characterized by low rates at some time points (such as gamma distribution at $t = 0$ for $\alpha > 1$), this approximation can be poor even in the limit of a large number of processes. MOSAIC circumvents this problem by setting $\lambda_{\max}$ to an arbitrary large value (for example $\lambda_{\max} \geq \lambda_0$), such that the time increment Δt remains *small enough* for the first order Taylor approximation (Eq. 87) to hold during the entire simulation. Still, this condition may not be sufficient when the number of processes N is too low (Supplementary Figure S2A). To handle these cases, we can set $\lambda_{\max}$ at each iteration such that:

$$\lambda_{\max} \geq \lambda_0 \cdot \min\left\{\frac{f}{N}, 1\right\}, \qquad [90]$$

where λ_0 is the inverse of the mean inter-event time distribution, and f a factor defined by the user to guarantee a desired upper bound for Δt, e.g. such as $\psi_j(t_j) \gg \psi_j'(t_j)\Delta t$ always holds:

$$\Delta t\ (\text{MOSAIC}) \leq \frac{\ln(1/u)}{f \cdot \lambda_0}. \qquad [91]$$

An important consideration is that this definition will affect the system only when the systems contains a low number of processes. For systems with a large number of processes ($N \gg f$), the relationship

$$\max_{\{j\in[1,N]\}} \lambda_j(t_j) \geq f \cdot \frac{\lambda_0}{N} \qquad [92]$$

is always verified with high probability, so that

$$\lambda_{\max} \geq \max_{\{j\in[1,N]\}} \lambda_j(t_j) \qquad [93]$$

regardless of the value of f. On the other hand, for systems with a low number of processes ($N < f$), increasing f results in increased accuracy, but at the cost of additional computational time. More precisely, the computational cost of running MOSAIC will increase from $O(rN)$ to $O(rf)$, where r is the attempted-over-accepted ratio of the system for $f = 1$. Using the general equation for the MOSAIC error (Eq. 79), and substituting $\lambda_{\max}$ from Eq. 90 we get that for $f \geq N$,

$$\text{Error}[\text{IED}_{\text{MOSAIC}}] \leq \frac{N \cdot \langle\lambda'\rangle}{f^2 \cdot \lambda_0^2}. \qquad [94]$$

and otherwise ($f < N$)

$$\text{Error}[\text{IED}_{\text{MOSAIC}}] \leq \frac{\langle\lambda'\rangle}{N \cdot \lambda_0^2}. \qquad [95]$$

Interestingly, our empirical investigation shows that such consideration only significantly affect the simulation accuracy when $N < 30$ (Supplementary Figure S2B & Figure S3), which falls well below the number of reactants typically involved in most practical scenarios. In particular, the choice f did not impact computational cost and accuracy the systems we discussed in this main article.

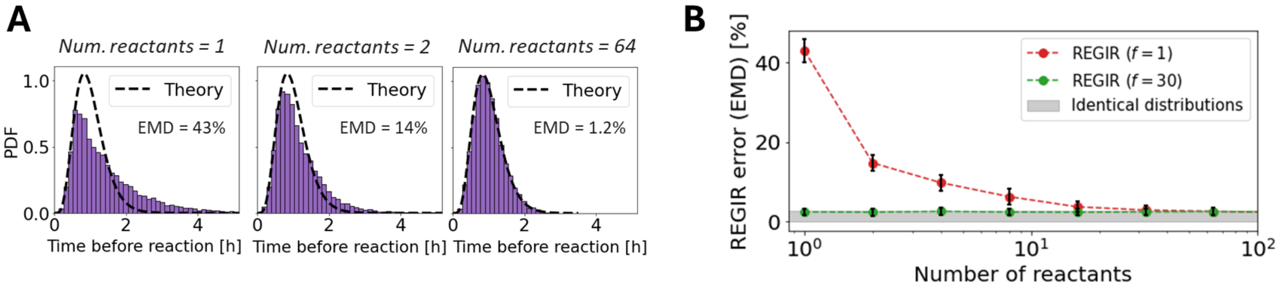

Figure S2: (A) MOSAIC ($f = 1$) approximation accuracy on a toy reaction $A \to \varnothing$ with a gamma inter-event distribution of shape parameter 6, visualized for different population sizes. The accuracy is computed using the earth mover distance (EMD) between the theoretical and simulated distributions, given in units of the distribution mean $1/\lambda_0$. In (B), we show how the EMD scales with the population size for two variations of MOSAIC, where the difference between the two lies in the additional parameter f used to define $\lambda_{\max}$.

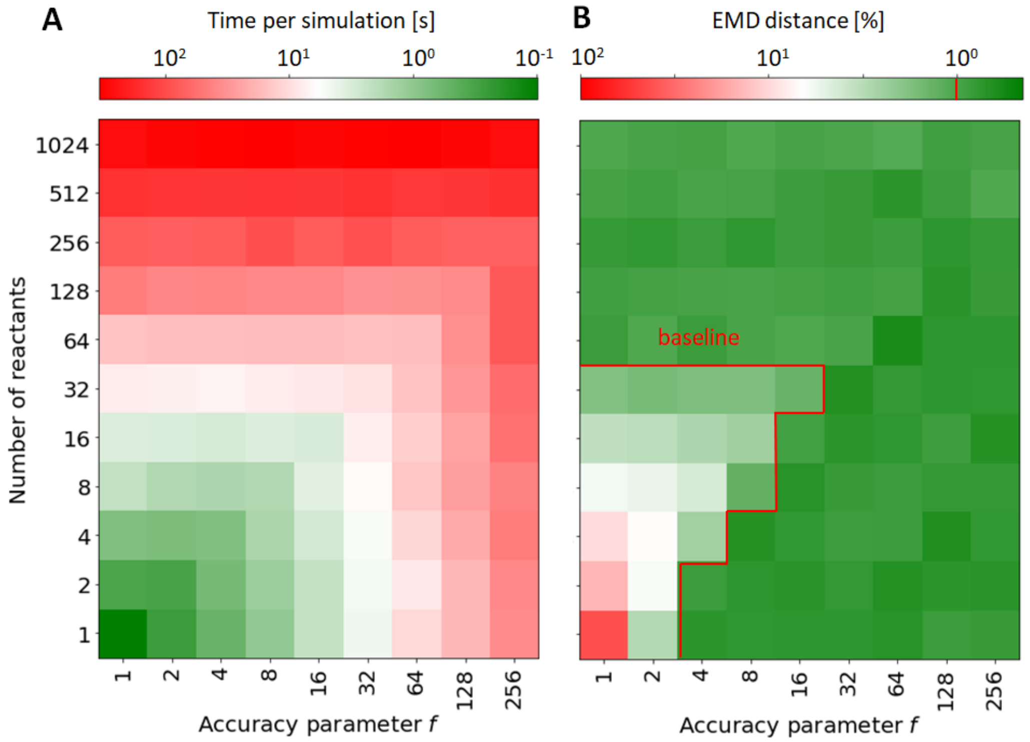

Figure S3: Trade-off between computational cost and accuracy. The toy reaction $A \to \varnothing$ is simulated with a gamma inter-event time distribution with parameters ($\lambda_0 = 1$, $\alpha = 6$). We display the heatmap of (A) the computational time for one simulation and (B) the EMD distance from the theoretical distribution. All values are averaged over 100 simulations, and EMD distances are given with a $\pm 10\%$ confidence interval. The red lines mark the border where the EMD becomes below the baseline EMD (defined as the EMD between two equal distributions with 10k sampling, equal here to 1%).

B.5 Rejection sampling reduces the computational complexity of MOSAIC to *O(N)*

We consider a simple reaction channel with N reactants and an arbitrary IED over a duration T. Let us examine two different aspects of this simulation: (i) the computational complexity of a single time step and its scaling properties, and (ii) the number of steps required to simulate a fixed interval of length T. Let us first focus on the scaling properties of a single-time step. During a large simulation, two factors contribute to its computational cost, the calculation of the time step to the next reaction, and the update of the reactant populations [13]. For SG with one reaction channel, both of these steps are $O(1)$. In the case of nMGA however, these updates carry a computational cost of $O(N)$, as each reactant has its own reaction rate, which is equivalent to having its own reaction channel. In particular, the update of the instantaneous rates can be very expensive, as it requires recalculating them for each process in the entire population after every simulation iteration. On the other hand, MOSAIC reduces the

computational complexity to $O(1)$ using a rejection base approach, where only the rate of the drawn reactant is computed at each step. The maximum rate $\lambda_{\max}$ is either kept constant throughout the simulation or is updated using an ordered data structure for storing the t_j values, thus also $O(1)$ (See Methods 3.1).

Regarding the number of steps required to simulate a fixed interval, SG, nMGA, Laplace, and DelaySSA scale linearly with the number of reactants N, because the time step becomes increasingly smaller as more channels are added to the simulation (Eq.2). However, the rejection approach of MOSAIC results in a larger number of time steps required to simulate a T_{end} interval, as a fraction of the time steps are rejected by the algorithm. The complexity then becomes $O(N) + O(R)$, where R refers to the number of rejected steps. From Eq.155 and Eq.4, we can compute the probability of rejection for a given iteration as:

$$
\begin{aligned}
p_{reject} &= 1 - p_{accept} \\
&= 1 - \frac{\sum_{j=1}^{N} p_j \lambda_j(t_j)}{\lambda_{\max}} \\
&= 1 - \frac{1}{N\lambda_{\max}} \sum_{j=1}^{N} \lambda_j(t_j) \\
&= 1 - \frac{\lambda_0}{\lambda_{\max}},
\end{aligned}
\tag{96}
$$

where λ_0 is the average observed rate. Then, the expected number of rejections before the first accepted reaction (A) is given by the mean of the geometric distribution with success probability p_{accept}:

$$
\frac{R}{A} = \frac{1}{p_{accept}} - 1 = \frac{(\lambda_{\max} - \lambda_0)}{\lambda_0}
\tag{97}
$$

Thus we can conclude that the scaling of SG and MOSAIC running times, $T_{\text{R, SG}}$ and $T_{\text{R, MOSAIC}}$, are proportional according to the relation:

$$
\frac{T_{\text{R, MOSAIC}}}{T_{\text{R, SG}}} = \frac{A + R}{A} = \frac{\lambda_{\max}}{\lambda_0}
\tag{98}
$$

Supplementary Figure S4 shows the rejected over accepted reactions ratio (R/A) for various distribution and shape parameters. We note that the ratio significantly varies with the choice of distribution and the parameters. For instance, an exponential distribution has a ratio of 0 (since $\hat{\lambda} = \lambda_{\max} = \lambda_i \; \forall i$, no reaction is rejected). On the other side of the spectrum, the Weibull instantaneous rate increases polynomially with time, so the maximum rate $\lambda_{\max}$ increases quickly with α, thus increasing the number of rejections. Other longer-tailed distributions with reaction rates increasing sub-linearly with time, e.g. the gamma distribution, will be less affected by changes in their respective shape parameter. In general, simulating a distribution with smaller variance will increase the maximum rate and as a result also increase the computational cost. This is intuitively clear from Supplementary Figure S4, where the rejected over accepted reaction ratio (R/A) monotonically increases with the shape parameter of the gamma and Weibull distributions, which inversely correlate to the variance of their respective distribution. On the other hand, as both R and A are proportional to the rate λ_0, the ratio R/A is independent of the mean IED $1/\lambda_0$ and thus scale invariant.

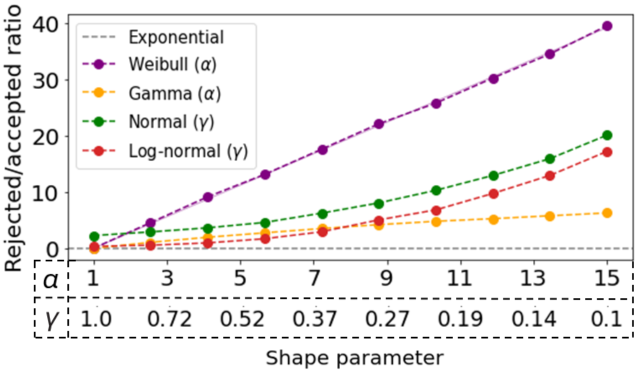

Figure S4: R/A ratio for different shape parameters of different distributions, averaged over 100 simulations of on a toy reaction $A \to \varnothing$. A standard deviation of $\sim \pm 12\%$ was observed for all measurements. Note that a direct comparison of the ratio across distributions is not meaningful as they have different standard deviations. We also note that R/A is scale-invariant and thus is independent of the distribution mean $1/\lambda_0$.

C Relationship between the node and the pairwise interactions IEDs

We consider two populations two type of reactants A and B, and a reaction channel **A + B** $\to$ **X**. When a reactant A_i interacts at a given time t, the probability of pairing with a specific reactant B_j is weighted by w_{B_j}, which represents B_j's internal state and history of interactions. Specifically, the probability of A_i to pair with B_j at time t is given by the ratio:

$$\frac{w_{B_j}(t)}{\sum_m w_{B_m}(t)}, \tag{99}$$

where the denominator ensures normalization across all potential partners B_m.

To compute the inter-event time distribution for the pair (A_i, B_j), we must account for all possible scenarios in which A_i does not pair with B_j for k consecutive interactions before finally pairing with B_j on the $(k+1)$-th interaction, which we write as:

$$P(\text{pairing with } B_j \text{ after } k \text{ steps}) = \prod_{i=1}^{k} \left(1 - \frac{w_{B_j}(t_i)}{\sum_m w_{B_m}(t_i)}\right) \cdot \frac{w_{B_j}(t_{k+1})}{\sum_m w_{B_m}(t_{k+1})}. \tag{100}$$

Here, the first term (the product) represents the probability that A_i did not pair with B_j during the first k interactions, while the second term represents the probability of pairing with B_j at the $(k+1)$-th interaction.

The inter-event time distribution for the pair (A_i, B_j), denoted $\psi_{AB}(t)$, is influenced by the activity pattern of A_i, represented by $\psi_A(t)$, and the probabilities of pairing (A_i, B_j) after k prior interactions. Specifically, it is expressed as a weighted sum of convolutions of $\psi_A(t)$, where the weights are determined by the pairing probabilities:

$$\psi_{AB}(t) = \sum_{k=0}^{\infty} \left[\prod_{i=1}^{k} \left(1 - \frac{w_{B_j}(t_i)}{\sum_m w_{B_m}(t_i)}\right) \cdot \frac{w_{B_j}(t_{k+1})}{\sum_m w_{B_m}(t_{k+1})}\right] \cdot \psi_A^{*(k+1)}(t), \tag{101}$$

where $\psi_A^{*(k+1)}(t)$ denotes the $(k+1)$-fold convolution of $\psi_A(t)$. This formulation highlights how the inter-event time distribution of (A_i, B_j) depends on both A_i's activity and the probabilistic nature of its pairings with B_j.

Assuming N_B a constant number of nodes over time, the term $\frac{w_{B_j}(t_i)}{\sum_m w_{B_m}(t_i)}$ can be approximated as $\frac{1}{N_B}$ on average. Under this assumption, the pairing probability simplifies, and the likelihood of A_i pairing with B_j after k prior interactions follows a geometric distribution:

$$P(\text{pairing with } B_j \text{ after } k \text{ steps}) = \frac{1}{N_B}\left(1 - \frac{1}{N_B}\right)^k. \tag{102}$$

As a result, the inter-event time distribution for (A_i, B_j) simplifies to:

$$\psi_{AB}(t) = \sum_{k=0}^{\infty} \frac{1}{N_B}\left(1 - \frac{1}{N_B}\right)^k \cdot \psi_A^{*(k+1)}(t). \tag{103}$$

As the mean of k convoluted distribution is the sum of their respective mean, i.e. $\mathbb{E}\left[\psi_A^{*(k)}\right] = k \cdot \mathbb{E}\left[\psi_A\right]$, and recognizing the derivative of a geometric series, we conclude using the linearity of the expected value that the means of the distributions $\psi_A(t)$ and $\psi_{AB}(t)$ are related by:

$$\mathbb{E}[\psi_{AB}] = N_B \cdot \mathbb{E}[\psi_A]. \tag{104}$$

For systems where the nodes' activity follows a Poisson process, i.e. $\psi_A(t) = \lambda_A \exp\left(-\lambda_A t\right)$, we can also write

$$\psi_{AB}(t) = \frac{\lambda_A}{N_B} \exp\left(-\frac{\lambda_A}{N_B} \cdot t\right). \tag{105}$$

For non-Poissonian activity patterns, however, the combination of convolutions and geometric probabilities makes $\psi_{AB}(t)$ challenging to compute explicitly, often requiring numerical methods or approximations even if we assume a uniform probability of selected B_j. Nevertheless, we can still make general statements about $\psi_{AB}(t)$, particularly when $\psi_A(t)$ exhibits long-tailed behavior. If $\psi_A(t)$ follows a heavy-tailed distribution, such as a power law, the convolution $\psi_A^{*(k+1)}(t)$ retains this heavy-tailed nature, with a progressively slower decay as k increases. This means that even without explicitly computing $\psi_{AB}(t)$, we know it will inherit the heavy-tailed characteristics of $\psi_A(t)$. Still, the link between the two distributions is not straightforward, and controlling one does not necessarily translate to control over the other. Given the complexity of the relationship between the inter-event time distribution for individual pairs and that of individual nodes in the network, it is crucial to clearly define and prioritize the specific aspect to focus on in the modeling process.

D MOSAIC for node-driven temporal networks (MOSAIC-TN)

D.1 Algorithm

Let us consider a reaction channel $\mathbf{A} + \mathbf{A} \to \mathbf{X}$. We denote by N_A the number of nodes. We first consider a fully connected topology, so we have $N = N_A(N_A - 1)/2$ processes. For each individual node A_i $(1 \leq i \leq N_A)$ and A_j $(1 \leq j \leq N_A)$, we consider t_i and t_j the time elapsed since the last interaction involving node A_i and A_j, respectively. At each iteration, two nodes A_i and A_j are drawn, and their interaction propensity is determined by a time-dependent pairwise interaction rate $\Lambda_{ij}(t_i, t_j) > 0$, which accounts for the individual times of both nodes. Importantly, Λ_{ij} can also incorporate pair-specific properties or interaction history of the node pair (i, j), enabling the modeling of *temporal neighborhood effects* , that is, interaction dynamics shaped by the past interactions or unique relationship between the two nodes. The MOSAIC algorithm for node-driven temporal networks (MOSAIC-TN) involves four steps:

(i) Set $\Lambda_{\max}$, the maximum reaction rate over all processes, such that:

$$\Lambda_{\max} \geq \max_{\{(i,j)\in[1,N_A],\ i\neq j\}} \Lambda_{ij}(t_i, t_j). \tag{106}$$

(ii) Compute the time increment to the next event using $\Lambda_{\max}$. Namely, a random variable is uniformly drawn from the interval $[0, 1]$, i.e. $u \in \mathcal{U}^{[0,1]}$, and the time increment is computed as:

$$\Delta t = \frac{2\ln(1/u)}{N_A(N_A - 1) \cdot \Lambda_{\max}}. \tag{107}$$

(iii) Select the two reactants A_i and B_j for the next event. All reactants have an equal probability of being drawn, and therefore, the probability of selecting A_i and A_j are:

$$p_i = \frac{1}{N_A} \text{ and } p_j = \frac{1}{N_A - 1}, \quad \text{respectively} \tag{108}$$

(iv) Accept the process with probability p_{accept}, given by:

$$p_{\text{accept}} = \frac{\Lambda_{ij}(t_i, t_j)}{\Lambda_{\max}}, \tag{109}$$

and update the reactants' population accordingly. If the process is rejected, the next event is set to an empty event, i.e. the reactant populations remain unchanged.

Here, the definition of the pairwise rate Λ_{ij} is crucial, as it directly governs both the interaction rate between node pairs and the resulting inter-event time distribution for individual nodes. We assume that, each node possesses an *intrinsic* instantaneous rate, denoted as $\lambda_i(t_i)$ and $\lambda_j(t_j)$ for nodes A_i and A_j, respectively. These rates, akin to activity-driven models, quantify the node's propensity to interact and can be conceptualized as a broadcast signal indicating its willingness to engage in interactions. We also introduce a scale free rate $\tilde{\lambda}(t) = \lambda(t)/\lambda_0$ and an arbitrary scaling constant Λ_0 for clarity.

For a given pair of nodes, we can consider different interaction scenarios. In one scenario, interactions are dominated by the most active node, reflecting an independent additive process. Here, the pairwise rate can be expressed as $\Lambda_{ij} \propto \Lambda_0 \left[\tilde{\lambda}_i(t_i) + \tilde{\lambda}_j(t_j)\right]$, making it well-suited for modeling directed communication networks, such as epidemic spreading, email exchanges or message transmissions. Alternatively, in a scenario where interactions require synchronous availability between the two nodes, a multiplication emerges as a natural choice, $\Lambda_{ij} \propto \Lambda_0 \left[\tilde{\lambda}_i(t_i) \cdot \tilde{\lambda}_j(t_j)\right]$, accounting for the fact that the interaction rate is limited by the node with the lower availability, as neither can sustain the interaction independently. This type of temporal system, requiring synchronization or simultaneous availability of interacting agents, is prevalent across various fields. In neuroscience, the precise timing of signals between neurons is critical for effective communication, as synaptic transmission often relies on the synchronized release of neurotransmitters and receptor activation [14]. Similarly, in social interactions, effective engagement typically requires both participants to be active simultaneously [15]. In molecular biology, ligand-receptor binding events also depend on synchronization, as successful interactions occur only when both molecules are available at the same time [16].

In Supplementary Section D.3, we explore different formulations of the pairwise interaction rate Λ_{ij} and their influence on the inter-event time distribution of individual nodes. With the additive rate, nodes can still engage in interactions even when their intrinsic rate is zero, as they may be randomly paired with a highly active node. As a result, there remains a nonzero probability of events occurring at $t_i \approx 0$, limiting the flexibility in shaping the inter-event time distribution. This constraint is particularly relevant for distributions such as Gamma or Weibull ($\alpha > 1$), which inherently assign zero probability to events occurring at $t = 0$. On the other hand, we prove in Supplementary Section D.4 that defining a multiplicative pairwise rate Λ_{ij} with

$$\Lambda_{ij}(t_i, t_j) = \frac{N_A}{N_A - 1} \cdot \frac{\lambda_i(t_i) \cdot \lambda_j(t_j)}{\sum_{k=1}^{N_A} \lambda_k(t_k)} \tag{110}$$

allows for the inter-event time distribution of node A_j to converge to the PDF of its intrinsic rate as $N_A \to \infty$, through the relation:

$$\psi_{Aj}(t) \underset{N_A \to \infty}{=} \lambda_j(t) \times \exp\left(-\int_0^t \lambda_j(\tau)\, d\tau\right). \tag{111}$$

The formulation of the multiplicative rate Λ_{ij}, emerges naturally from considering the minimum event times of N_A independent processes, governed by a total rate of $\sum_{k=1}^{N_A} \lambda_k(t_k)$ (Supplementary Section E). It reflects the combined likelihood of both nodes being available, weighted by their respective contributions to the total interaction rate. The additional term $N_A/(N_A - 1)$ is a correction factor to account for the fact that a node cannot interact with itself, as derived in Supplementary Section D.4.

D.2 Approximation of the mean observed rate

While computing the term $N_A \langle \lambda \rangle = \sum_{k=1}^{N_A} \lambda_k(t_k)$ may increase computational cost due to the need to iterate over all nodes at each step, it can be approximated by introducing the mean observed instantaneous rate, $\lambda_0 = \frac{1}{N_A} \left\langle \sum_{k=1}^{N_A} \lambda_k(t_k) \right\rangle$. Using this approximation, Λ_{ij} can be expressed as:

$$\Lambda_{ij}(t_i, t_j) = \frac{N_A}{N_A - 1} \cdot \frac{\lambda_i(t_i) \cdot \lambda_j(t_j)}{\left\langle \sum_{k=1}^{N_A} \lambda_k(t_k) \right\rangle} \approx \frac{\lambda_i(t_i) \cdot \lambda_j(t_j)}{(N_A - 1) \cdot \lambda_0}. \tag{112}$$

In Supplementary Section F, we show that, in the steady state, the mean observed rate is given by $\lambda_0 = \frac{1}{N_A} \sum_{i=1}^{N_A} \lambda_{0_i}$, where $\lambda_{0_i} = 1/\mathbb{E}[\tau_i]$, and $\mathbb{E}[\tau_i]$ denotes the mean inter-event time of the intrinsic PDF for each individual node,

$$\mathbb{E}[\tau_i] = \int_0^\infty t \cdot \psi_{Aj}(t)\, dt. \tag{113}$$

D.3 Choice of the pairwise interaction rate function Λ_{ij}

We provide here the mathematical guarantees underlying MOSAIC-TN, as well as the rationale behind our choice of the pairwise rate formula. Here we consider a system in which each node, belonging to class A or B, follows its own intrinsic rate function, $\lambda_A(\tau_A)$ and $\lambda_B(\tau_B)$, respectively, and that their pairwise interaction rate is given by $\Lambda(\tau_A, \tau_B)$. Here, τ_A and τ_B represent the internal times of reactants A and B, respectively.

Effective inter-event time distributions: Let ψ_A^{eff} and ψ_B^{eff} denote the *effective* PDF of inter-event time distribution of individual reactants A and B, respectively. These PDFs are referred to as effective because they cannot be directly expressed as functions of the intrinsic rates $\lambda_A(t)$ and $\lambda_B(t)$, due to the coupling introduced by the interaction term Λ. These effective PDFs differ from the PDFs the reactants A and B in isolation $\psi_A(t) = \lambda_A(t) \cdot \exp(-\int_0^\infty \lambda_A(t))$ and $\psi_B = \lambda_B(t) \cdot \exp(-\int_0^\infty \lambda_B(t))$, which we refer to as the *intrinsic* PDFs.

In the general case, the effective inter-event time distribution for reactant A is determined by the minimum inter-event time over all possible interactions with B, which corresponds to the combined instantaneous rates of all such interactions, represented by the sum $\sum_{k=1}^{N_B} \Lambda(\tau_A, \tau_{Bk})$. Note that, while we focus here on the effective dynamics of reactant A, the same formulation can be applied to B symmetrically without loss of generality. The effective SDF for A, denoted as $\Psi_A^{\text{eff}}(t)$, can thus be expressed as:

$$\Psi_A^{\text{eff}}(t) = \exp\left(-\int_0^t \sum_{k=1}^{N_B} \Lambda(\tau_A, \tau_{Bk})\, d\tau_A\right), \quad \text{where} \quad \tau_{Bk} \sim \psi_B^{\text{obs}}(\tau_B) \qquad [114]$$

represents the internal times of the k-th reactant B, sampled according to the *observed* PDF $\psi_B^{\text{obs}}(\tau_B)$. Formally, $\psi_B^{\text{obs}}(\tau_B)$ is the PDF of the backward recurrence time associated with $\psi_B^{\text{eff}}(\tau_B)$, as we discuss in Supplementary Section F.

Poisson processes. The simplest case arises when the rates of both reactants, λ_A and λ_B, are constant. In this scenario, for any pairwise rate function Λ, the effective survival function of A is given by

$$\Psi_A^{\text{eff}}(t) = \exp\left(-N_B \int_0^t \Lambda\Big(\lambda_A, \lambda_B\Big)\, d\tau\right) = e^{-N_B \cdot \Lambda\left(\lambda_A, \lambda_B\right) \cdot t}. \qquad [115]$$

If we account for individual properties within the population of B, where each individual reactant B_k has its own rate λ_{Bk}, the observed inter-event time distribution for A is influenced by the heterogeneity in B's rates. In this case, the effective survival function for A reflects the cumulative contributions of all interactions with B_k, and is given by

$$\Psi_A^{\text{eff}}(t) = e^{-\sum_{k=1}^{N_B} \Lambda\left(\lambda_A, \lambda_{Bk}\right) \cdot t}. \qquad [116]$$

.

Multiplicative rates: For multiplicative rate, $\Lambda(\tau_A, \tau_B) = c \cdot \lambda(\tau_A) \cdot \lambda(\tau_B)$, we can write

$$\Psi_A^{\text{eff}}(t) = \exp\left(-c \int_0^t \sum_{k=1}^{N_B} \lambda(\tau_{Bk}) \cdot \lambda(\tau_A)\, d\tau_A\right), \qquad [117]$$

$$\Psi_A^{\text{eff}}(t) = \exp\left(-c \sum_{k=1}^{N_B} \lambda(\tau_{Bk}) \int_0^t \lambda(\tau_A)\, d\tau_A\right), \qquad [118]$$

$$[119]$$

Defining the mean observed rate $\langle \lambda_B \rangle = \frac{1}{N_B} \sum_{k=1}^{N_B} \lambda(\tau_{Bk})$, we can write

$$\Psi_A^{\text{eff}}(t) = \exp\left(-\int_0^t \lambda(\tau_A)\, d\tau_A\right)^{cN_B \cdot \langle \lambda_B \rangle} \qquad [120]$$

$$\Psi_A^{\text{eff}}(t) = \Big[\Psi_A(t)\Big]^{cN_B \cdot \langle \lambda_B \rangle} \qquad [121]$$

Finally, we can conclude that, by setting $c = 1/(N_B \cdot \langle \lambda_B \rangle)$, we always have $\Psi_A^{\text{eff}}(t) = \Psi_A(t)$.

Additive rates: For additive rate, $\Lambda(\tau_A, \tau_B) = c \cdot [\lambda(\tau_A) + \lambda(\tau_B)]$, we can write

$$\Psi_A^{\text{eff}}(t) = \exp\left(-c\int_0^t \sum_{k=1}^{N_B} \lambda(\tau_A) + \lambda(\tau_{Bk})\, d\tau_A\right), \quad [122]$$

$$\Psi_A^{\text{eff}}(t) = \exp\left(-cN_B\int_0^t \lambda(\tau_A)\, d\tau_A\right) \cdot \exp\left(-cN_B\int_0^t \langle\lambda_B\rangle\, d\tau_A\right) \quad [123]$$

$$\Psi_A^{\text{eff}}(t) \approx \Big[\Psi_A(t)\Big]^{cN_B} \cdot \exp\Big(-cN_B\langle\lambda_B\rangle \cdot t\Big) \quad [124]$$

Setting the constant $c = 1/N_B$, we obtain

$$\Psi_A^{\text{eff}}(t) \approx \Big[\Psi_A(t)\Big] \cdot e^{-\langle\lambda_B\rangle t}, \quad [125]$$

from which we derive the effective PDF as

$$\psi_A^{\text{eff}}(t) \approx \left[\Psi_A(t)\right] \cdot \langle\lambda_B\rangle e^{-\langle\lambda_B\rangle t} + \psi_A(t) \cdot e^{-\langle\lambda_B\rangle t}. \quad [126]$$

Interestingly, this is the mixture of two PDFs. The first behave like an exponential distribution for low time, since $\Psi_A(0) = 1$, while the second is the intrinsic PDF but scaled with an additional exponential tail.

D.4 The reaction A_i + A_j $\rightarrow$ X

In the particular case of two reactants of the same type reacting with each others, the MOSAIC algorithm is slightly modified. The reaction $\mathrm{A} + \mathrm{A} \rightarrow X$ involve $\frac{1}{2}N_A(N_A - 1)$ processes, and the probabilities of choosing reactants A_i and A_j at each iteration are given by $\frac{1}{N_A}$ and $\frac{1}{N_A-1}$, respectively. In this case, the observed SDF is directly related to the observed PDF with

$$\Psi_A^{\text{eff}}(t) = \exp\left(-\int_0^t \sum_{k=1}^{N_A-1} \Lambda(\tau_A, \tau_{Ak})\, d\tau_A\right), \quad \text{where} \quad \tau_{Ak} \sim \psi_A^{\text{obs}}(\tau_A) \quad [127]$$

That summation includes the reaction rates of $N_A - 1$ nodes, excluding the node of interest A. In the case of the multiplicative rate, $\Lambda(\tau_{A_i}, \tau_{A_j}) = c \cdot \lambda(\tau_{A_i}) \cdot \lambda(\tau_{A_j})$, we can write

$$\Psi_A^{\text{eff}}(t) = \exp\left(-c\sum_{k=1}^{N_A-1} \lambda(\tau_{Ak})\int_0^t \lambda(\tau_A)\, d\tau_A\right), \quad [128]$$

Defining the mean observed rate $\langle\lambda_A\rangle = \frac{1}{N_A}\sum_{k=1}^{N_A} \lambda(\tau_{Ak})$, we have

$$\sum_{k=1}^{N_A-1} \lambda(\tau_{Ak}) = \sum_{k=1}^{N_A} \lambda(\tau_{Ak}) - \lambda(\tau_A) = N_A\langle\lambda_A\rangle - \lambda(\tau_{Ak}) \quad [129]$$

and we can thus rewrite

$$\Psi_A^{\text{eff}}(t) = \exp\left(-\int_0^t \lambda(\tau_A)\, d\tau_A\right)^{c\cdot[N_A\langle\lambda_A\rangle - \lambda(\tau_{Ak})]}. \quad [130]$$

Defining c as

$$c = \frac{N_A}{N_A - 1} \cdot \frac{1}{N_A \cdot \langle\lambda_A\rangle}, \quad [131]$$

we get an effective SDF

$$\Psi_A^{\text{eff}}(t) = \left[\exp\left(-\int_0^t \lambda(\tau_A)\, d\tau_A\right)\right] \wedge \left(\frac{N_A - \frac{\lambda(\tau_A)}{\langle\lambda_A\rangle}}{N_A - 1}\right) \underset{N_A\to\infty}{=} \Psi_A(t), \quad [132]$$

which converge to the intrinsic density distribution for large N_A, as the factor involving self reaction becomes diluted by the number of processes and becomes negligible.

D.5 MOSAIC-TN on constrained topologies

In many applications, interactions are constrained by an underlying static network (e.g., a contact graph), so that only a subset of all node pairs can interact. Let N_A denote the number of nodes and let $\mathcal{E}$ be the set of admissible unordered node pairs (i,j) consistent with the prescribed topology. We write $N = |\mathcal{E}|$ for the number of such pairs; in the fully connected case, $\mathcal{E}$ contains all node pairs and $N = N_A(N_A - 1)/2$.

Algorithmically, MOSAIC-TN extends straightforwardly to this setting. We first choose an upper bound

$$\Lambda_{\max} \;\geq\; \max_{(i,j)\in\mathcal{E}} \Lambda_{ij}(t_i,t_j), \tag{133}$$

over all admissible pairs. Drawing $u \sim \mathcal{U}[0,1]$, the time increment to the next candidate event is now

$$\Delta t = \frac{\ln(1/u)}{N\,\Lambda_{\max}}, \tag{134}$$

which reduces to Eq. [107] when the topology is fully connected and $N = N_A(N_A-1)/2$. A candidate interaction is then obtained by sampling a pair (i,j) uniformly from $\mathcal{E}$, i.e. with probability $1/N$, and accepting it with probability

$$p_{\text{accept}} = \frac{\Lambda_{ij}(t_i,t_j)}{\Lambda_{\max}}, \tag{135}$$

as in Eq. [109]. This rejection-based construction still yields $O(1)$ computational complexity per candidate event.

The choice of pairwise rate Λ_{ij} and the associated guarantees follow the same logic as in the fully connected case. For a given node i with neighbor set $\mathcal{N}(i)$, the effective survival function of its inter-event time is

$$\Psi_i^{\text{eff}}(t) = \exp\left(-\int_0^t \sum_{j\in\mathcal{N}(i)} \Lambda_{ij}(\tau_i,\tau_j)\,d\tau_i\right). \tag{136}$$

Under the same mean-field assumptions used in Sections D.3 and D.4, a multiplicative rate of the form

$$\Lambda_{ij}(t_i,t_j) \propto \lambda_i(t_i)\,\lambda_j(t_j), \tag{137}$$

with an appropriate normalization (replacing the global factor N_A by the local degree $|\mathcal{N}(i)|$ and the global mean rate by a local average over neighbors), ensures that $\Psi_i^{\text{eff}}(t)$ converges to the intrinsic survival function $\Psi_i(t)$ for nodes with sufficiently large degree. In particular, the marginal node-level IEDs retain their imposed shape (including heavy-tailed behavior), while the constrained topology introduces heterogeneity in the rate at which nodes experience events. Thus, MOSAIC-TN preserves its mathematical guarantees on node-level inter-event distributions even when interactions are restricted to arbitrary sparse or structured topologies.

E Derivation of the Pairwise Interaction Rate for Poisson Processes requiring Synchronous Availability

We consider N_A nodes, indexed by $k = 1, 2, \ldots, N_A$, each emitting events according to independent Poisson processes with instantaneous rates λ_k. Let T_k denote the time until the next event from node k. The next pairwise interaction occurs at the minimum of these event times, $T = \min(T_1, T_2, \ldots, T_N)$. The total rate of events occurring at time T, Λ_{total}, is derived from the property of the *minimum of independent exponential random variables*. Specifically, if $T_k \sim \text{Exp}(\lambda_k)$ for all k, then $T = \min(T_1, T_2, \ldots, T_N)$ is itself exponentially distributed with a rate equal to the sum of the individual rates:

$$\Lambda_{\text{total}} = \sum_{k=1}^{N_A} \lambda_k. \tag{138}$$

This result arises because the survival probability of the minimum is the product of the survival probabilities of the individual random variables:

$$P(T > t) = \prod_{k=1}^{N_A} P(T_k > t) = \prod_{k=1}^{N_A} e^{-\lambda_k t} = e^{-\left(\sum_{k=1}^{N_A} \lambda_k\right)t}. \tag{139}$$

For an interaction between two nodes i and j to occur, both nodes must contribute by aligning their events. The probability that node i contributes the next event is proportional to its rate, given by:

$$P(T = T_i) = \frac{\lambda_i}{\sum_{k=1}^{N_A} \lambda_k}, \quad [140]$$

and similarly, the probability that node j contributes is:

$$P(T = T_j) = \frac{\lambda_j}{\sum_{k=1}^{N_A} \lambda_k}. \quad [141]$$

The pairwise interaction rate, Λ_{ij}, is the product of the total rate of events, Λ_{total}, and the joint probability of contributions from i and j. This joint probability is proportional to the likelihood that both nodes emit events aligned in time:

$$\Lambda_{ij} = \Lambda_{\text{total}} \cdot P(T = T_i) \cdot P(T = T_j). \quad [142]$$

Substituting the expressions for Λ_{total}, $P(T = T_i)$, and $P(T = T_j)$, we have:

$$\Lambda_{ij} = \left(\sum_{k=1}^{N_A} \lambda_k \right) \cdot \frac{\lambda_i}{\sum_{k=1}^{N_A} \lambda_k} \cdot \frac{\lambda_j}{\sum_{k=1}^{N_A} \lambda_k}. \quad [143]$$

Simplifying, this yields the effective pairwise interaction rate:

$$\Lambda_{ij} = \frac{\lambda_i \cdot \lambda_j}{\sum_{k=1}^{N_A} \lambda_k}. \quad [144]$$

F Observed backward recurrence time and instantaneous rates

We consider a renewal process described by an arbitrary probability density function (PDF) $\psi_A(\tau)$, a survival density function $\Psi_A(\tau)$, and its instantaneous rate $\lambda_A(\tau) = \psi_A(\tau)/\Psi_A(\tau)$. When observing this process at a random point in time, the elapsed time τ since the last event corresponds to the *backward recurrence time* in renewal theory [17, 18], which represents the time elapsed since the most recent renewal event. The PDF of the backward recurrence time, $\psi_A^{\text{obs}}(\tau)$, is given by [17]

$$\psi_A^{\text{obs}}(\tau) = \frac{\Psi_A(\tau)}{\mathbb{E}[\tau]} = \lambda_0 \Psi_A(\tau), \tag{145}$$

where we define λ_0 the reciprocal of the expected elapsed time $\mathbb{E}[\tau]$, defined as:

$$\lambda_0 = \frac{1}{\mathbb{E}[\tau]} = \left(\int_0^\infty t \cdot \psi_A(\tau)\, d\tau \right)^{-1}. \tag{146}$$

Specifically, the observed backward recurrence time does not follow the original PDF $\psi_A(t)$; instead, it is weighted by the survival of intervals, as longer intervals are more likely to be observed. This phenomenon, commonly referred to as the *length-biased sampling effect*, is frequently discussed in studies related to disease screening [19].

In the context of MOSAIC, our focus is on the rate observed at each iteration, as it directly governs the probability of an event being accepted. The *mean observed rate* can be computed by integrating the instantaneous rate $\lambda_A(\tau)$, weighted by the observed PDF $\psi_A^{\text{obs}}(\tau)$:

$$\mathbb{E}_{\text{obs}}[\lambda_A(\tau)] = \int_0^\infty \lambda_A(\tau) \cdot \psi_A^{\text{obs}}(\tau)\, d\tau. \tag{147}$$

Substituting $\psi_A^{\text{obs}}(\tau) = \lambda_0 \Psi_A(\tau)$, the expression becomes:

$$\mathbb{E}_{\text{obs}}[\lambda_A(\tau)] = \lambda_0 \int_0^\infty \lambda_A(\tau) \cdot \Psi_A(\tau)\, d\tau. \tag{148}$$

By noting that $\lambda_A(\tau) \cdot \Psi_A(\tau) = \psi_A(\tau)$, the integral simplifies to:

$$\mathbb{E}_{\text{obs}}[\lambda_A(\tau)] = \lambda_0 \int_0^\infty \psi_A(\tau)\, d\tau. \tag{149}$$

Since $\int_0^\infty \psi_A(\tau)\, d\tau = 1$, as $\psi_A(\tau)$ is a probability density function, the mean observed rate reduces to:

$$\mathbb{E}_{\text{obs}}[\lambda_A(\tau)] = \lambda_0. \tag{150}$$

Now, we extend this result to N independent renewal processes, each characterized by its own PDF $\psi_{A_i}(\tau)$, survival function $\Psi_{A_i}(\tau)$, and mean rate $\lambda_{0_i} = 1/\mathbb{E}[\tau_i]$. The observed PDF for randomly sampling a node and observing its rate is determined by the average contribution of all processes

$$\psi_A^{\text{obs}}(\tau) = \frac{1}{N} \sum_{i=1}^{N} \lambda_{0_i} \Psi_{A_i}(\tau). \tag{151}$$

Substituting this expression into Eq. 147 and following the same reasoning as in the single-process case, we find:

$$\mathbb{E}_{\text{obs}}[\lambda_A(\tau)] = \frac{1}{N} \sum_{i=1}^{N} \lambda_{0_i}. \tag{152}$$

G Non uniform selection of processes in MOSAIC

Motivation. The rejection approach in MOSAIC is particularly beneficial when individual rates are expensive to compute and fluctuate with each iteration. However, when handling a wide array of individual rates, this method can slow down the simulation, as processes with low rates are frequently rejected [6]. This issue is notably significant in processes involving pairs of reactants. In such cases, a small number of pairs with high interaction rates can disproportionately slow down the system, while many pairs with zero propensity still undergo selection and subsequent rejection. If these zero-propensity pairs can be identified before each iteration without intensive computation, it is more efficient to exclude them from selection altogether, rather than selecting and then rejecting them as MOSAIC does.

Here, we introduce a modified version of MOSAIC that adjusts the probability of selecting certain processes over others, thereby reducing the number of rejections up to several orders of magnitudes. This modification ensures that processes with highly disparate rates are handled more efficiently, and processes with zero rates are excluded from selection entirely.

Algorithm. We denote by t_j the time elapsed since the last event of the jth process $(1 \leq j \leq N)$, and by $\lambda_j(t_j)$ the time-dependent reaction rate of the jth process. At each iteration, the modified MOSAIC performs 4 steps:

(i) Set $\lambda_{\max}$, the maximum reaction rate over all processes, such that:

$$\lambda_{\max} \geq \max_{\{j \in [1,N]\}} \lambda_j(t_j). \quad [153]$$

(ii) Compute the time increment to the next event as in SG using $\lambda_{\max}$. Namely, a random variable is uniformly drawn from the interval $[0,1]$, i.e. $u \in \mathcal{U}^{[0,1]}$. The time increment is computed as:

$$\Delta t = \frac{\ln(1/u)}{N \cdot \lambda_{\max}}. \quad [154]$$

(iii) Assign a weight w_j to each process, ensuring that the sum of all weights $\sum_{k=1}^{N} w_k = N$. Then, select the process j that has triggered the event, with the probability of selecting each process being

$$p_j = \frac{w_j}{N}. \quad [155]$$

(iv) Accept the process with probability p_{accept}, given by:

$$p_{\text{accept}} = \frac{\lambda_j(t_j)}{\lambda_{\max}}, \quad [156]$$

and update the reactants' population accordingly. If the process is rejected, the next event is set to an empty event, i.e. the reactant populations remain unchanged.

This algorithm's output resembles the original MOSAIC, with the difference that it scales the intrinsic rate of each process by w_j:

$$\lambda_j^{\text{obs.}}(t_j) = w_j \cdot \lambda_j(t_j), \quad [157]$$

Importantly, This modification affects the computational cost of the simulation compared to using the original MOSAIC. Denoting by M the number of unique weight $M = |set_{j \leq N}\{w_j\}|$, step (iii) now has a complexity of $O(M)$ instead of $O(1)$ as in the original MOSAIC framework. Consequently, the computational complexity of simulating the system becomes $\mathrm{O}(r'MN)$, with the key observation that the ratio of attempted to accepted reactions r' is now lower than the ratio when all weights were equal r. Therefore, employing this modified version of MOSAIC is advantageous when the number of different weights to consider is less than the computational savings from reduced rejections, i.e. $M > r/r'$.

We note that scaling the rate by a weight at each iteration will affect the obtained distribution differently depending on the rate function. In the case of the exponential distribution, this is simply equivalent to scaling the inter-event time distribution of the jth process by w_j, i.e. $\lambda_{j0}^{\text{obs.}} = w_j \cdot \lambda_{j0}$. Similarly, if $\lambda_j(t_j)$ follows a Weibull distribution, then this is also equivalent to scaling the distribution by w_j, with its shape parameter α remaining unchanged. Finally, if $\lambda_j(t_j)$ adheres to a Pareto distribution, the obtained distribution will also be a Pareto but the weights will affect both the rate and the shape of the distribution. For most other rate functions however, the resulting distribution does not follow a simple form.

Proof that modified MOSAIC follow the same distribution as Standard Gillespie. To prove each processes in the weighted MOSAIC follows $\lambda_j^{\text{obs.}}(t_j) = w_j \cdot \lambda_j$, we take a similar approach as we did in Supplementary Section B.1. ie. we prove that:

(i) The reaction R_j occurs with probability $p = w_j\lambda_j / \sum w_j \lambda_j$

We define $p_{\text{accept}}(R_j)$ as the joint probability of R_j being first selected and then accepted, $\lambda_{\max} \geq \max_{\{j \in [1,N]\}} \lambda_j$ as the upper propensity bound of all reactions , and $a_{0,\max} = N\lambda_{\max}$. We can write

$$p_{\text{accept}}(R_j) = \frac{w_j}{N} \cdot \frac{\lambda_j}{\lambda_{\max}} = \frac{w_j \lambda_j}{a_{0,\max}}$$

We then denote by $p_{\text{accept}}(R)$ the probability of any reaction being accepted:

$$p_{\text{accept}}(R) = \frac{1}{N} \sum w_j \cdot \frac{\lambda_j}{\lambda_{\max}} = \frac{\sum w_j \lambda_j}{a_{0,\max}}.$$

The conditional probability $p_{\text{accept}}(R_j \mid R)$ can be exploited to show the probability of R_j being accepted given that some reaction had been accepted:

$$p_{\text{accept}}(R_j \mid R) = \frac{p_{\text{accept}}(R_j)}{p_{\text{accept}}(R)} = \left(\frac{w_i \lambda_j}{a_{0,\max}}\right) / \left(\frac{\sum w_j \lambda_j}{a_{0,\max}}\right) = \frac{w_i \lambda_j}{\sum w_j \lambda_j}$$

(ii) The Δt increment in time follows the same exponential distribution as in SG, i.e. $f_{\Delta t}(t) \sim \sum w_j \lambda_j \cdot \exp(-\sum w_j \lambda_j \cdot t)$

Here the derivation is exactly the same as in Supplementary Section B.1. Given that $p_{\text{accept}}(R) = \frac{\sum w_j \lambda_j}{a_{0,\max}}$, we obtain $\Delta t \sim (\sum w_j \lambda_j) \exp(-a_{0,\max} \cdot t) \cdot \exp(x \cdot (a_{0,\max} - \sum w_j \lambda_j)) = (\sum w_j \lambda_j) \cdot \exp(-\sum w_j \lambda_j \cdot t)$.

H Computational complexity of stochastic algorithms

In this section, we provide an analysis of the computational complexity of various stochastic simulation algorithms.

- **Gillespie Algorithm:** The standard Gillespie algorithm has a known computational complexity of $O(M)$ per reaction step, where M is the number of unique reaction rates. This complexity arises from the necessity of drawing a reaction based on propensity values at each iteration. Specifically, the reaction index μ is selected by searching for the smallest μ satisfying

$$\sum_{j=1}^{\mu-1} a_j < r_2 \sum_{j=1}^{M} a_j, \leq \sum_{j=1}^{\mu} a_j. \qquad [158]$$

- **Tree-Based Gillespie Algorithm** [20]: By utilizing a binary tree structure, the selection process can be improved from $O(M)$ to $O(\log M)$. In this approach, reaction propensities are stored in a binary tree, where each node maintains a partial sum of the propensities of its subtree. Reaction selection is then performed using a binary search, reducing the selection time to $O(\log M)$. However, since the tree needs to be updated whenever reaction propensities change, its efficiency depends on the system. Specifically, each update requires modifying the relevant nodes along the path from the updated leaf to the root, leading to an update complexity of $O(k \log M)$, where k is the number of updated reactions per step. For systems where propensities remain largely unchanged throughout the simulation, the tree-based approach can be highly efficient. Conversely, for systems with rapidly changing rates, maintaining the tree can be computationally expensive, sometimes outweighing the benefits over the standard Gillespie algorithm.

- **Laplace Gillespie Algorithm** [5]: The Laplace Gillespie algorithm extends the standard Gillespie algorithm to non-Markovian processes by incorporating non-exponential inter-event times. Instead of redrawing all N rates at each step, it samples them initially from $p(\lambda)$ and updates only the selected reaction's rate thereafter. To efficiently handle selection and updates, the algorithm employs a tree-based structure where reaction propensities are stored hierarchically, enabling $O(\log N)$ selection via binary search and efficient updates. This structure maintains the algorithm's efficiency while allowing for flexible non-Markovian dynamics.

- **nMGA (Non-Markovian Gillespie Algorithm)** [7]: This method requires recomputing all N reaction rates at each time step, making tree-based approaches inefficient. The overall complexity per iteration is thus $O(N)$, dominated by the full rate update.

- **Delayed Stochastic Simulation Algorithm (DelaySSA)** [21]: This algorithm maintains a sorted list of scheduled events, allowing the next event to be extracted in $O(1)$ time. However, maintaining the sorted structure incurs an additional cost of $O(k \log N)$ per iteration, where k is the number of events to be updated. New events are inserted at the correct position using binary search, followed by an efficient insertion operation in a balanced tree or linked structure [22]. Deletion is always performed at the front of the list in $O(1)$ time.

For stochastic simulations on temporal networks, where interactions occur among N nodes, there are N^2 potential interactions, or processes, to consider.

- **Standard Gillespie Algorithm on Temporal Networks:** Since interactions take place between pairs of nodes, each with potentially different rates, the number of processes to track per time step is $O(N^2)$. Consequently, the total computational complexity for a full simulation scales as $O(N^4)$.

- **Spanning Tree Approach** [15]: A spanning tree is constructed to model temporal interactions, ensuring that nodes and links follow predefined IED. The initial construction of the spanning tree incurs a computational cost of $O(N^2)$, given that each node interacts with a significant approximatly half of the other nodes at least once. During the simulation, interactions evolve over d time steps, with each step requiring updates based on the IED distributions. In the worst case, all edges must be considered at each step, leading to a total computational complexity of $O(dN^2)$.

- **Activity-Driven (AD) Modeling** [23]: At each time step, node pairs are assessed to determine potential interactions. Most activity-driven modeling studies consider complexities such as historical dependencies and heterogeneous activity levels when defining interactions [24], requiring iteration over all possible interactions. Given that the process unfolds over d time steps, the overall computational complexity is $O(dN^2)$.

Supplementary Figures

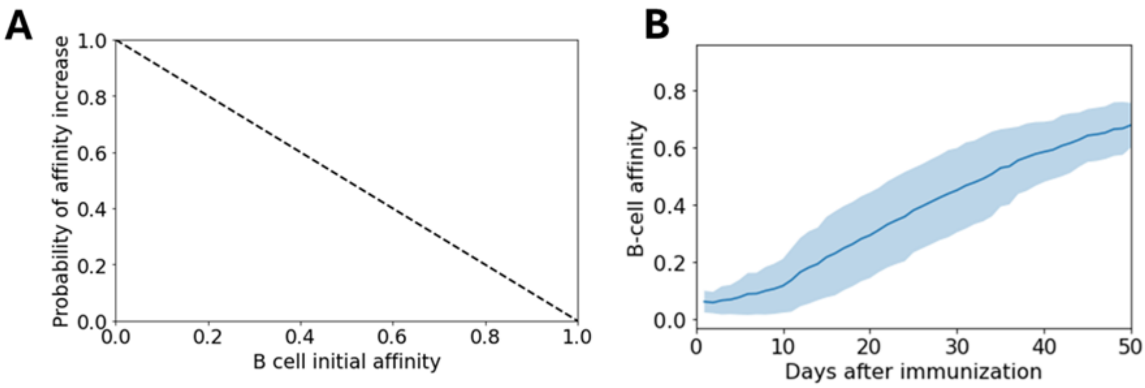

Figure S5: (A) Probability of the daughter cell increasing the affinity from the parent cell as a function of the initial affinity. (B) Affinity change of GC B cells during a germinal center simulation. The line is the average over all B cells and the shaded area represent the mean plus one positive and one negative standard deviation.

Bibliography

[1] Renyan Jiang and DNP Murthy. “A study of Weibull shape parameter: Properties and significance”. In: *Reliability Engineering & System Safety* 96.12 (2011), pp. 1619–1626.

[2] William E Boyce, Richard C DiPrima, and Douglas B Meade. *Elementary differential equations*. John Wiley & Sons, 2017.

[3] Walter Gautschi. “Error function and Fresnel integrals”. In: *Handbook of mathematical functions* 55 (1972), pp. 297–308.

[4] John T Sauls et al. "Control of Bacillus subtilis replication initiation during physiological transitions and perturbations". In: *MBio* 10.6 (2019), e02205–19.

[5] Naoki Masuda and Luis EC Rocha. "A Gillespie algorithm for non-Markovian stochastic processes". In: *SIAM Review* 60.1 (2018), pp. 95–115.

[6] Vo Hong Thanh, Corrado Priami, and Roberto Zunino. "Efficient rejection-based simulation of biochemical reactions with stochastic noise and delays". In: *The Journal of chemical physics* 141.13 (2014), 10B602_1.

[7] Marian Boguná et al. "Simulating non-Markovian stochastic processes". In: *Physical Review E* 90.4 (2014), p. 042108.

[8] Brook Taylor. *Methodus incrementorum directa & inversa*. Inny, 1717.

[9] Harry H Ku et al. "Notes on the use of propagation of error formulas". In: *Journal of Research of the National Bureau of Standards* 70.4 (1966).

[10] Patrick S Stumpf et al. "Stem cell differentiation as a non-Markov stochastic process". In: *Cell Systems* 5.3 (2017), pp. 268–282.

[11] Jennifer C England et al. "Global regulation of gene expression and cell differentiation in Caulobacter crescentus in response to nutrient availability". In: *Journal of bacteriology* 192.3 (2010), pp. 819–833.

[12] Amit Zeisel et al. "Coupled pre-mRNA and mRNA dynamics unveil operational strategies underlying transcriptional responses to stimuli". In: *Molecular systems biology* 7.1 (2011), p. 529.

[13] Kevin R Sanft and Hans G Othmer. "Constant-complexity stochastic simulation algorithm with optimal binning". In: *The Journal of chemical physics* 143.7 (2015), 08B609_1.

[14] Ryan C Kelly and Robert E Kass. "A framework for evaluating pairwise and multiway synchrony among stimulus-driven neurons". In: *Neural computation* 24.8 (2012), pp. 2007–2032.

[15] Anzhi Sheng et al. "Constructing temporal networks with bursty activity patterns". In: *Nature Communications* 14.1 (2023), p. 7311.

[16] J Alberto Guevara-Salazar et al. "Use of the harmonic mean to the determination of dissociation constants of stereoisomeric mixtures of biologically active compounds". In: *Journal of Enzyme Inhibition and Medicinal Chemistry* 29.6 (2014), pp. 884–894.

[17] Paul D Allison. "Survival analysis of backward recurrence times". In: *Journal of the American Statistical Association* 80.390 (1985), pp. 315–322.

[18] Marvin Zelen. "Forward and backward recurrence times and length biased sampling: age specific models". In: *Lifetime Data Analysis* 10 (2004), pp. 325–334.

[19] Stephen W Duffy et al. "Correcting for lead time and length bias in estimating the effect of screen detection on cancer survival". In: *American journal of epidemiology* 168.1 (2008), pp. 98–104.

[20] Michael A Gibson and Jehoshua Bruck. "Efficient exact stochastic simulation of chemical systems with many species and many channels". In: *The journal of physical chemistry A* 104.9 (2000), pp. 1876–1889.

[21] Xiaoming Fu et al. "DelaySSAToolkit. jl: stochastic simulation of reaction systems with time delays in Julia". In: *Bioinformatics* 38.17 (2022), pp. 4243–4245.

[22] James O Henriksen. "Event list management-a tutorial". In: *Proceedings of the 15th conference on Winter Simulation-Volume 2*. 1983, pp. 543–551.

[23] Nicola Perra et al. "Activity driven modeling of time varying networks". In: *Scientific reports* 2.1 (2012), p. 469.

[24] Didier Le Bail, Mathieu Génois, and Alain Barrat. "Modeling framework unifying contact and social networks". In: *Physical Review E* 107.2 (2023), p. 024301.
